# Diversity of root hydrotropism among natural variants of *Arabidopsis thaliana*

**DOI:** 10.1007/s10265-022-01412-w

**Published:** 2022-09-23

**Authors:** Boyuan Mao, Hiroki Takahashi, Hideyuki Takahashi, Nobuharu Fujii

**Affiliations:** Graduate School of Life Sciences, Tohoku University, 2-1-1 Katahira, Aoba-Ku, Sendai, 980-8577 Japan

**Keywords:** Arabidopsis, Natural variants, Root hydrotropism, Root gravitropism

## Abstract

Root gravitropism affects root hydrotropism. The interference intensity of root gravitropism with root hydrotropism differs among plant species. However, these differences have not been well compared within a single plant species. In this study, we compared root hydrotropism in various natural variants of Arabidopsis under stationary conditions. As a result, we detected a range of root hydrotropism under stationary conditions among natural Arabidopsis variants. Comparison of root gravitropism and root hydrotropism among several Arabidopsis natural variants classified natural variants that decreased root hydrotropism into two types; namely one type that expresses root gravitropism and root hydrotropism weaker than Col-0, and the other type that expresses weaker root hydrotropism than Col-0 but expresses similar root gravitropism with Col-0. However, root hydrotropism of all examined Arabidopsis natural variants was facilitated by clinorotation. These results suggested that the interference of root gravitropism with root hydrotropism is conserved among Arabidopsis natural variants, although the intensity of root gravitropism interference with root hydrotropism differs.

**Supplementary Information:**

The online version contains supplementary material available at 10.1007/s10265-022-01412-w.

## Introduction

Plants are sessile in nature. Therefore, it is thought that plants have obtained tropic responses through evolutionary processes to adapt to environmental conditions by changing the growth directions of their own organs in response to environmental stimuli. After germination, roots bend downward by positive root gravitropism and enter the ground to absorb nutrients and water from soils (review in Nakamura et al. 2019; Vandenbrink and Kiss 2019). Roots can also express positive root hydrotropism, bending toward higher water-potential directions (review in Dietrich 2018; Takahashi 1997; Takahashi et al. 2009). Root gravitropism can interfere with root hydrotropism, namely, under stationary conditions, roots of agravitropic pea mutants can express hydrotropism, although roots of wild-type pea cannot express hydrotropism (Jaffe et al. 1985). This conclusion was confirmed by the induction of root hydrotropism in wild-type pea by clinorotation of seedlings (Takahashi et al. 1996). Similarly, root hydrotropism of cucumber could be expressed by clinorotation, although a stationary condition could not induce hydrotropism in cucumber roots (Mizuno et al. 2002). In contrast to pea and cucumber, *Arabidopsis thaliana* (L.) Heynh. (Arabidopsis), lotus and rice can express root hydrotropism under stationary conditions (Nakajima et al. 2017; Takahashi et al. 2002). These results indicated that the strength of the interference of root gravitropism on root hydrotropism differs among plant species.

The Cholodny-Went hypothesis has been widely accepted to explain gravitropism, and this hypothesis is composed of three steps: (1) relocalization of auxin efflux carriers to the lower side of gravity-sensing cells (statocytes) is induced by placement of plants in a horizontal position (gravistimulation), (2) the polar-localized auxin efflux carriers induce asymmetric distribution of auxin across organs by asymmetric transport of auxin, and (3) auxin responses to asymmetrically distributed auxin cause asymmetrical cell elongation so that organs bend (review in Nakamura et al. 2019; Vandenbrink and Kiss 2019). Asymmetric transport of auxin has been suggested to be involved in root hydrotropism in cucumber seedlings because auxin-inducible genes are asymmetrically expressed during root hydrotropism, inhibitors of auxin efflux carriers such as TIBA and HFCA inhibit root hydrotropism, and auxin efflux carriers (CsPIN5) accumulate more in the epidermal cells in the higher moisture side than in the lower moisture side of cucumber roots (Fujii et al. 2018; Mizuno et al. 2002; Morohashi et al. 2017). Root hydrotropism in rice and pea was also prevented by inhibitors of auxin efflux carriers, suggesting that auxin transport plays a role in expressing root hydrotropism in rice and pea roots (Nakajima et al. 2017). In contrast, it has been shown that Arabidopsis and lotus do not require auxin transport to express root hydrotropism (Kaneyasu et al. 2007; Nakajima et al. 2017; Shkolnik and Fromm 2016; Takahashi et al. 2002).

To reveal the molecules that are required for root hydrotropism, genetic analysis using Arabidopsis has been conducted, revealing *mizu-kussei1* (*miz1*) and *miz2* mutants with impaired root hydrotropism that still express root gravitropism (Kobayashi et al. 2007; Miyazawa et al. 2009). MIZ1 proteins localize to the endoplasmic reticulum, but the molecular function of MIZ1 protein is still unknown (Yamazaki et al. 2012). *miz2* mutation was identified in the *GNOM* gene, which encodes a guanine nucleotide exchange factor of ARF GTPase (ARF-GEF) and is responsible for PIN localization by regulating the intracellular trafficking pathway, although *miz2* mutation did not affect PIN localization (Miyazawa et al. 2009; Moriwaki et al. 2014). *gnom* mutants were originally isolated as embryo lethal mutants or defective mutants in the establishment of the embryonic axis (Mayer et al. 1993; Shevell et al. 1994). In contrast to the *miz2* mutant, partial loss-of-function alleles of *gnom* mutants impaired not only hydrotropism but also gravitropism as well as postembryonic development (Geldner et al. 2004; Moriwaki et al. 2014). Additionally, treatment with brefeldin A (BFA), which inhibits the activities of ARF-GEFs, including GNOM, inhibits root gravitropism as well as root hydrotropism in Arabidopsis (Geldner et al. 2001; Miyazawa et al. 2009). These results implied that *miz2* mutation affects the hydrotropism-specific intracellular trafficking pathway. In addition, it has been revealed that abscisic acid (ABA) signals play a role in root hydrotropism in Arabidopsis (Belda-Palazon et al. 2018; Dietrich et al. 2017; Takahashi et al. 2002). In contrast to ABA, it has been shown that reduced auxin responses by auxin antagonists accelerate hydrotropism in Arabidopsis roots (Shkolnik et al. 2016).

Miao et al. (2018) showed that there is a difference in hydrotropic responses among Arabidopsis ecotypes or accessions, namely, that the hydrotropic curvature of C24, which is an Arabidopsis ecotype, was significantly less than those of Columbia (Col-0) and Wassilewskija (Ws). Comparative transcriptome analysis among C24, Col-0 and Ws suggested the possibility that brassinosteroids affect the expression of root hydrotropism (Miao et al. 2018). However, the number of natural variants of which root hydrotropism was examined was insufficient to understand the diversity and/or the conserved nature of the relationship between root gravitropism and root hydrotropism within one plant species. Therefore, in this study, we examined the root hydrotropism of various natural variants of Arabidopsis and found the diversity of root hydrotropism among various natural variations of Arabidopsis.

## Materials and methods

### Plant material and growth conditions

Seeds of Arabidopsis accessions and T-DNA mutants were purchased from ABRC (Arabidopsis Biological Resource Center). Seeds were surface-sterilized with 5% (v/v) sodium hypochlorite and 0.05% (v/v) Tween 20 for 5 min and were placed on half strength Murashige and Skoog medium (0.23% (w/v) MS salts (Fujifilm Wako Pure Chemical, Osaka, Japan), 0.5% (w/v) sucrose, and 0.03% (w/v) gellan gum (Sigma-Aldrich, MO, USA), pH 5.8). After seeds for sowing were stored at 4 °C for 2 days in the dark, plates were placed in a vertical position under continuous light conditions at 22 °C to allow the seedlings to grow onto the surface of the medium.

### Tropism assays and measurement of root curvature

**Fig. 1:**
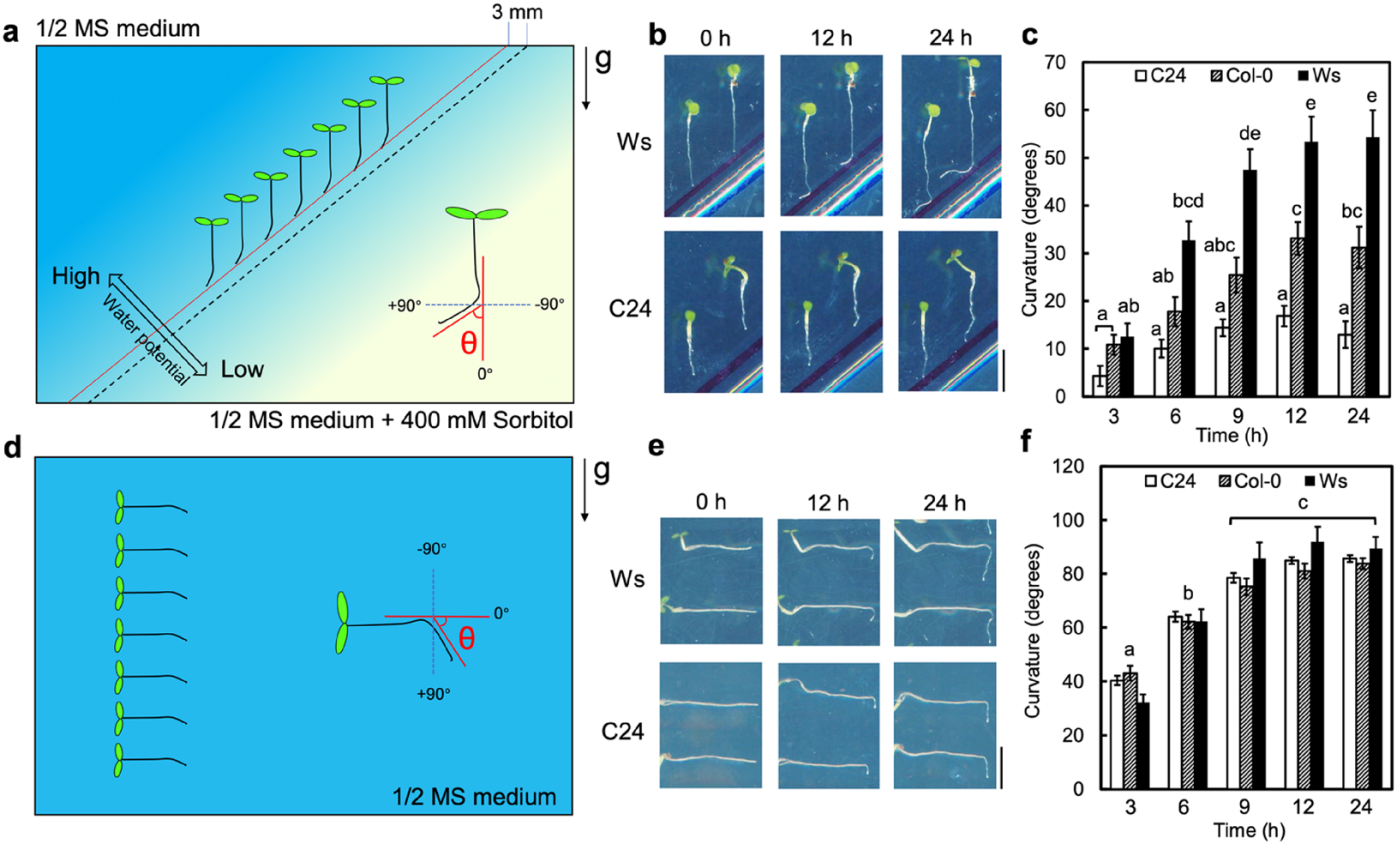
Comparison of hydrotropism and gravitropism of Col-0, Ws and C24 roots. **a** Split-agar assay system for the study of hydrotropism. Water-potential gradients were induced by combining 1% agar and 1% agar containing 400 mM sorbitol in a square plastic dish. The four-day-old seedlings were placed on 1% agar, where the root tip was 3 mm from the border between 1% agar and 1% agar containing 400 mM sorbitol. On the bottom right side in the drawings, a schematic representation of how the hydropic curvature was measured is shown. **b** Ws and C24 seedlings after exposure to hydrotropic stimulation for 0, 12, and 24 h. **c** Time course of hydrotropic curvatures of Col, Ws and C24 roots. **d** Experimental system for the study of gravitropism. The four-day-old seedlings were reoriented by 90° counterclockwise. On the right side in the drawings, a schematic representation of how the gravitropic curvature was measured is shown. **e** Ws and C24 seedlings after replacement in a 90° counterclockwise position for 0, 12, and 24 h. **f** Time course of the gravitropic curvature of Col-0, Ws and C24 roots. The curvatures **c** and **f** were measured after exposing seedlings to hydrotropic or gravitropic stimulation for 3, 6, 9, 12, and 24 h. White bars, C24; hatched bars, Col-0; black bars, Ws. Each data point is the average of three independent experiments that were conducted using 8–10 individuals per experiment. Error bars represent SEs. The arrow (g) indicates the direction of gravitational force. Scale bars, 5 mm

Four-day-old seedlings were used for all tropism assays. Hydrotropism assays were performed by using a modified split-agar assay system (Fig. 1a; Takahashi et al. 2002). Water-potential gradients were established between 1% agar plates and 1% agar plates containing 400 mM sorbitol (Fujifilm Wako Pure Chemical). Gravitropism assays were performed according to a previously described method (Kobayashi et al. 2007). The four-day-old seedlings were arranged vertically on 1% agar plates and then reoriented at an angle of 90° counterclockwise immediately before incubation at 22 °C in the dark (Fig. 1d). Roots were scanned by viewing from the back of the plate using an image scanner (CanoScan 9100F; Canon, Tokyo, Japan) after incubation for 0, 3, 6, 9, 12, and 24 h. Root curvature was measured by ImageJ software (NIH; http://rsb.info.nih.gov/ij). Statistical analysis was performed by Tukey’s HSD test using R studio software (http://www.rstudio.com/).

### Clinorotation hydrotropism assay

After seedlings were transplanted to the split-agar assay system, seedlings were immediately loaded on the clinostat, and the axles connected to the outer and inner frames were rotated at 1 rpm in the dark at 22 °C. Roots were scanned using an image scanner (CanoScan 9100F) after incubating for 12 and 24 h. Root curvature was measured by ImageJ software. Statistical analysis was performed by Tukey’s HSD test using R studio software.

### Genome-wide association studies (GWAS)

Using quantitative data of 24-h primary root hydrotropic curvature under stationary conditions of 217 Arabidopsis accessions, we conducted a GWAS using a simple linear regression (LM) (https://gwas.gmi.oeaw.ac.at/) (Seren et al. 2012).

## Results

### The split-agar system is appropriate for hydrotropism assays of Arabidopsis natural variants

To explore the hydrotropism among natural variants of Arabidopsis, we used a split-agar assay system (Fig. 1a). Water potential gradients were formed by combining a 1% agar medium and a 1% agar medium containing 400 mM sorbitol because plain medium has higher water potential and medium containing 400 mM sorbitol has lower water potential. In the presence of a water gradient, roots that were positioned in a vertical position bent toward the higher water potential side. The bending angle indicates the strength of the root hydrotropism ability. It has been shown that there are differences in hydrotropic curvatures among natural Arabidopsis variants or accessions such as Col-0, C24 and Ws (Miao et al. 2018). Prior to examining the root hydrotropism of various natural Arabidopsis variants, we compared root hydrotropism among Col-0, C24 and Ws by measuring the hydrotropic curvature after exposing seedlings to hydrotropic conditions for 0, 3, 6, 9, 12, and 24 h (Fig. 1b, c). The hydrotropic curvature after exposure to hydrosimulation for 12 and 24 h was C24 < Col-0 < Ws (Fig. 1b, c). After exposure to hydrosimulation for 24 h, the growth direction of some C24, Col-0 and Ws roots hydrotropic curvature seemed to be slightly reduced compared with that after exposure for 12 h although statistical analysis did not show differences between 12 and 24 h. Therefore, we decided to compare hydrotropic curvatures among Arabidopsis natural variants after hydrosimulation for 24 h.

We also compared the time-course of gravitropic bending responses among Col-0, C24 and Ws. Gravitropic stimulation was achieved by 90° rotation after transplanting seedlings to the plain medium (Fig. 1d). Compared with the bending of root hydrotropism, the bending of root gravitropism is faster. The gravitropic curvatures of C24, Col-0 and Ws roots already began to nearly plateau after exposure to gravitropic stimulation for 9 h, and it became difficult to distinguish differences among C24, Col-0 and Ws roots after 24 h (Fig. 1f). From these results, we decided to compare the gravitropic curvatures among several natural variants after exposure to gravistimulation for 12 h in later experiments.

### Hydrotropism among Arabidopsis natural variants changes gradually under stationary conditions

**Fig. 2:**
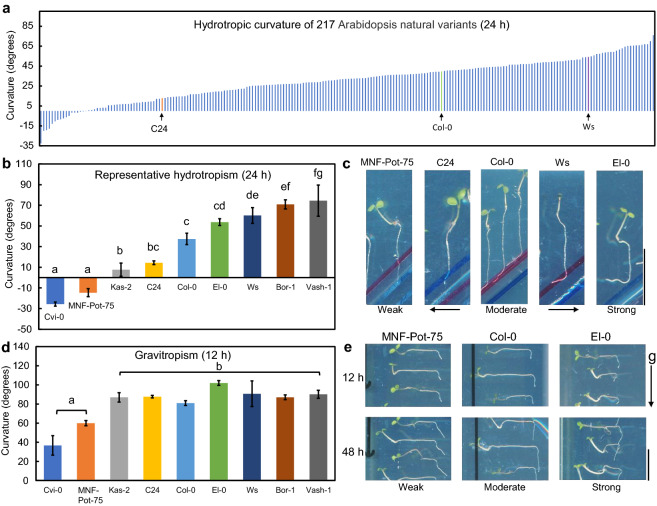
Root hydrotropic and gravitropic curvatures among natural variations of Arabidopsis. **a** Twenty-four-hour hydrotropic curvatures under stationary conditions of 217 Arabidopsis accessions. Each data point is the average of assays of 5–10 individuals. **b** Root hydrotropic curvature of representative natural variants of Arabidopsis after exposure to hydrosimulation for 24 h. **c** Representative natural variants of Arabidopsis that showed weak, moderate, and strong root hydrotropic responses after exposure to hydrosimulation for 24 h. **d** Root gravitropic curvature of representative natural variants of Arabidopsis after exposure to gravistimulation for 12 h. **e** Root gravitropic curvature of representative natural variants of Arabidopsis that showed weak, moderate, and strong root hydrotropic responses after exposure to gravistimulation for 12 and 48 h. **b** and **d** Each data point is the average of three independent experiments that were conducted using 8–10 individuals per experiment. Error bars represent SEs. Different letters indicate statistically significant differences (*P* < 0.05) with Tukey’s honestly significant difference (HSD) test. The arrow (g) indicates the direction of gravitational force. Scale bars, 10 mm

To examine the diversity of the interaction between root hydrotropism and root gravitropism in Arabidopsis, we screened 217 natural Arabidopsis variants with the split-agar assay system under stationary conditions (Fig. 2a) and quantified the primary root hydrotropic curvature (Table S1). As a result, the hydrotropic curvatures of 43 natural variants were less than that of C24, and those of 24 natural variants were greater than that of Ws.

As shown in Fig. 2b, c, we selected and examined representative natural variants to confirm reproductivity after harvesting the seeds. The root hydrotropic curvature among these 9 representatives gradually changed. In particular, Cvi-0 and MNF-Pot-75 almost lost their hydrotropism under stationary conditions. These results showed that hydrotropism among Arabidopsis natural variations changes gradually under stationary conditions. We also compared the root gravitropic curvatures of the 9 representatives. As shown in Fig. 2d, the gravitropic curvatures of Cvi-0 and MNF-Pot-75 roots were also significantly less than those of the other seven natural variants. Even after exposure to gravistimulation for 48 h, their roots did not bend completely in the direction of gravity (Fig. 2e). In contrast to Cvi-0 and MNF-Pot-75, the other seven selected natural variants exhibited similar root gravitropic curvatures (Fig. 2d).

To investigate the relationship between the root growth and root-bending angle of the 9 representatives, we blotted the growth length on the *x*-axis, the bending angle on the *y*-axis and analyze the correlation (Fig. S1). As a result, there is almost no correlation between root growth and root curvature of either root hydrotropism or root gravitropism (Fig. S1cd). Especially the root growth of Kas-2 during hydrotropism was the greatest (Fig. S1a), but hydrotropic bending of Kas-2 roots under stationary conditions was just slight (Fig. 2b). Therefore, the small bending angle seems to be not due to the greater growth inhibition by osmotic stress. In contrast, we found that root growth during root hydrotropism was relatively correlated with root growth during root gravitropism (*R*^2^ = 0.75; Fig. S1e). Therefore, it seems that differences in the root growth abilities among the natural variants do not depend on which tropism is expressed.

### Root gravitropism interferes with hydrotropism in Arabidopsis

**Fig. 3:**
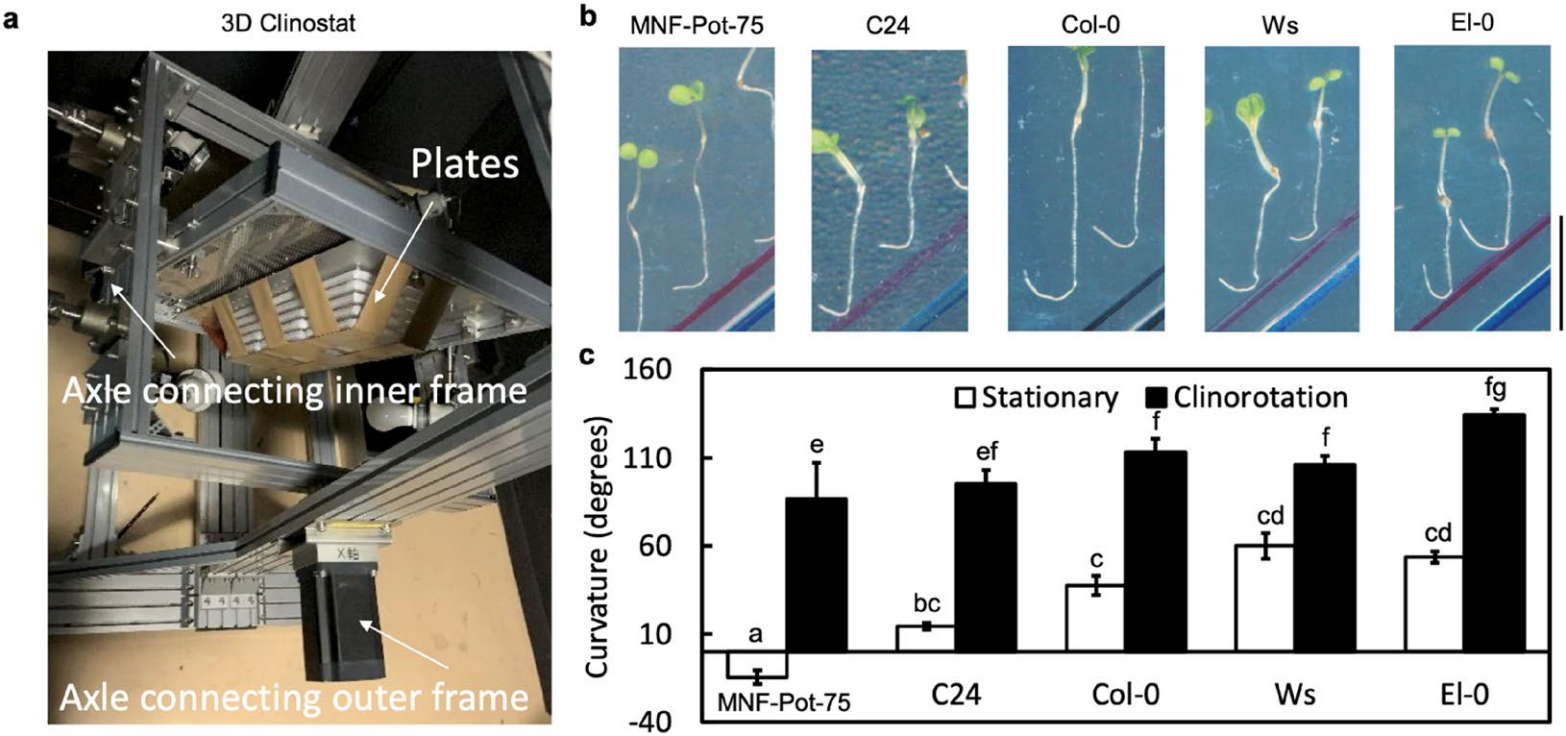
Root hydrotropic responses under clinorotation. **a** The mechanical configuration of the 3D clinostat. For the experiments, the axles connected to the outer and inner frames were rotated at 1 rpm. **b** Representative natural variants of Arabidopsis after exposure to hydrotropic stimulation for 24 h with clinorotation. **c** Effects of clinorotation on root hydrotropism of representative natural variants of Arabidopsis. Root hydrotropic curvatures of representative natural variants of Arabidopsis were measured after exposure to hydrosimulation in stationary conditions or under clinorotation for 24 h. Each data point is the average of three independent experiments that were conducted using 8–10 individuals per experiment. Error bars represent SEs. Different letters indicate statistically significant differences (*P* < 0.05) by Tukey’s honestly significant difference (HSD) test. Scale bar, 10 mm

To explore the interaction between hydrotropism and gravitropism, we examined several representatives via a split-agar assay system on a 3D clinostat (Fig. 3a). This device changes the direction of plants against the gravity vector by uniformly rotating the plants on the two axles that are in an orthogonal position, simulating microgravity conditions (Hoson et al. 1997). As shown in Fig. 3b, after exposure to hydrotropic stimulation with clinorotation for 24 h, the five representative roots strongly bent according to the direction of the high-water potential gradient. Compared with the root curvature induced by hydrotropic stimulation under stationary conditions, seedling curvature under clinorotation increased significantly among the five representatives (Fig. 3c). These results suggest that the interference of root gravitropism with root hydrotropism is conserved among Arabidopsis variants.

### Genome-wide association study (GWAS)

**Fig. 4:**
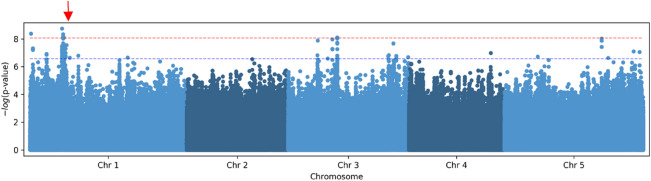
Manhattan plot of GWAS results of root hydrotropism of 217 Arabidopsis accessions under stationary conditions. The horizontal red dotted line and blue dotted line correspond to a significance level of 0.05 with Bonferroni and Bonferroni–Hochberg corrections for multiple tests, respectively. The red arrow indicates the position of *MYB52* gene that contains significant nonsynonymous polymorphisms and that was examined in this study

**Fig. 5:**
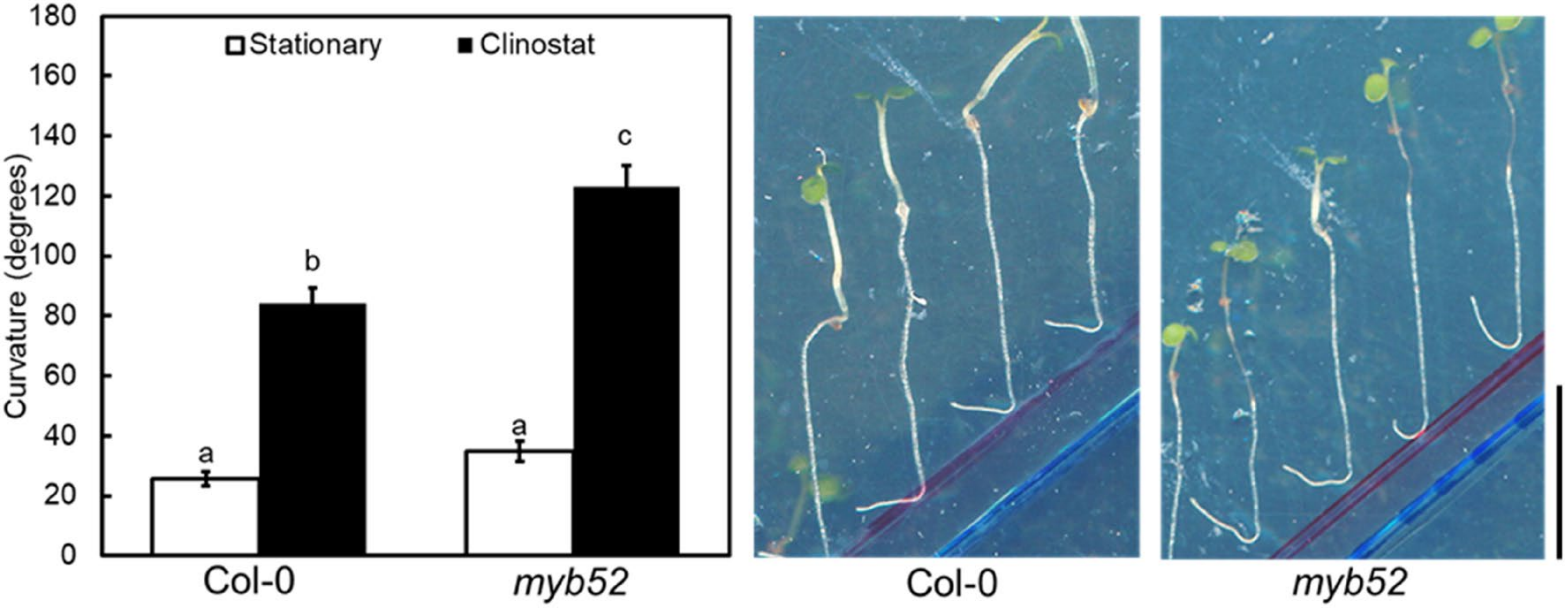
Root hydrotropic curvatures of Arabidopsis T-DNA mutants. Left, root hydrotropic curvatures of Col-0 and *myb52* seedlings were measured after exposure to hydrosimulation in stationary conditions or under clinorotation for 24 h. Each data point is the average of three independent experiments that were conducted using 8–10 individuals per experiment. Error bars represent SEs. Different letters indicate statistically significant differences (*P* < 0.05) with Tukey’s honestly significant difference (HSD) test. Right, Col-0 and *myb52* seedlings after exposure to hydrostimulation for 24 h under clinorotation. Scale bar, 10 mm

We attempted to identify the molecular participants involved in the diversity of root hydrotropism among natural Arabidopsis variants. To achieve this, we used quantitative data of 24 h primary root hydrotropic curvatures under stationary conditions and conducted a genome-wide association study (GWAS). We identified polymorphisms in several chromosomal regions that showed correlations with our characteristics when we used simple linear regression (LM) for GWAS (Fig. 4). Among those, several significant nonsynonymous polymorphisms that cause amino acid substitutions were found. If the nonsynonymous polymorphisms can affect root hydrotropism under stationary conditions, a T-DNA knockout mutation of the gene that contains the nonsynonymous polymorphisms will also be expected to affect root hydrotropism. In this study, we focused on AT1G17950 that has a significant nonsynonymous polymorphism in our GWAS (Fig. 4 and Fig. S2). AT1G17950 was named *MYB52* (Romero et al. 1998). First, we confirmed T-DNA insertion in *MYB52* gene of *myb52* mutant (SALK_138624C) by PCR (Fig. S3). And then, we examined the root hydrotropism in *myb52* mutant. Under stationary conditions, the root hydrotropism of *myb52* mutant did not significantly differ from that of Col-0 (Fig. 5). However, when the mutants were exposed to hydrostimulation with clinorotation, *myb52* mutant roots were more bent than Col-0 roots (Fig. 5). These results suggested the possibility that *MYB52* products play a role in hydrotropic bending responses. Therefore, our GWAS results possibly identified nonsynonymous polymorphisms that affect the interaction between root hydrotropism and root gravitropism.

## Discussion

### Experimental system to examine root hydrotropism

To investigate root hydrotropism in Arabidopsis, the combination of plain agar and agar containing chemicals of low water potential, such as sorbitol, is commonly used to induce a gradient of water potential (Li et al. 2020; Takahashi et al. 2002). In particular, two methods are most commonly used. One is the obliquely directed gradient of water potential that was used in this study, and the other is the vertically directed gradient of water potential that was described in Miao et al. (2018). The vertically directed gradient of water potential is consistent with the root growth direction and could not induce unidirectional curvature. Instead of unidirectional curvature, the inhibition of root elongation has been used to determine hydrotropism (Miao et al. 2018). However, the inhibition of root elongation differs from hydrotropic curvature. Therefore, a phenomenon detected by the experimental system with a vertically directed water potential gradient should differ from hydrotropism. Using the vertically directed gradient of water potential, inhibition of root elongation was observed as Cvi-0/C24 > Col-0 > Ws (Miao et al. 2018). Hydrotropic curvature that was induced by the obliquely directed gradient of water potential in this study was observed as Cvi-0 < C24 < Col-0 < Ws (Fig. 2b). This antiparallel relationship implied that more sensitive variants to low water potential show reduced hydrotropic responses. However, it should be noted that the root hydrotropic curvature of Cvi-0 was less than that of C24, although the inhibition of root elongation of Cvi-0 on the vertically directed gradient of water potential was not significantly different from that of C24. Therefore, this antiparallel relationship seems to not always hold true.

### Phylogenetic relationship of the interaction between root hydrotropism and root gravitropism

The strength of the root gravitropism interference with root hydrotropism differs among plant species. Nakajima et al. (2017) reported that rice and lotus roots displayed a hydrotropic response capable of overcoming their gravitropism. Arabidopsis, used in this study, also expresses root hydrotropism under stationary conditions (Takahashi et al. 2002). These results suggest that the root gravitropism of Arabidopsis, rice and lotus is relatively weaker than hydrotropism. However, it has been shown that the root gravitropism of cucumber and pea was stronger than the hydrotropism (Mizuno et al. 2002; Takahashi et al. 2009). Fig. S4 shows the relationship between plant linage and the intensity of root gravitropism/hydrotropism in plants. It has been shown that rice and Arabidopsis show a “root gravitropism < root hydrotropism” pattern and that cucumber shows a “root gravitropism > root hydrotropism” pattern. Therefore, if ancient plants had “root gravitropism < root hydrotropism” patterns because rice and Arabidopsis display this pattern and if the change from “root gravitropism < root hydrotropism” to “root gravitropism > root hydrotropism” occurred only one time, the relationship of “root gravitropism < root hydrotropism” would have changed to “root gravitropism > root hydrotropism” before cucumber evolutionally appeared. As a result, Fabales should display the “root gravitropism > root hydrotropism” relationship. However, Fabales contains lotus, which displays a “root gravitropism < root hydrotropism” dynamic, as well as pea, in which “root gravitropism > root hydrotropism”. Therefore, the relationship between root gravitropism and root hydrotropism should have been changed by two or more events. Namely, the strength between root hydrotropism and gravitropism does not depend on lineage differentiation, and the relationship between root hydrotropism and root gravitropism seems to have varied several times throughout evolution. This conclusion was strongly supported by differences in root hydrotropism under stationary conditions among Arabidopsis natural variants in this study.

It has been shown that gravitropism interferes with hydrotropism in pea and cucumber roots as well as Arabidopsis Col-0 roots (Jaffe et al. 1985; Kobayashi et al. 2007; Mizuno et al. 2002; Takahashi et al. 1996). In this study, even though differences in root hydrotropism under stationary conditions were observed among Arabidopsis natural variants, removing the influence of gravity responses through 3D clinorotation significantly increased the hydrosimulation response (curvature) of each natural variant (Fig. 3b, c). It is worth noting that even the natural variants that expressed little hydrotropic response under stationary conditions, such as MNF-Pot-75, were able to recover the hydrotropic response under clinorotation (Fig. 3b, c). Therefore, whether in a natural variant with strong or weak hydrotropism, gravitropism significantly interferes with root hydrotropism. Because the magnitude and direction of gravity in nature are not easy to change, gravitropism as well as the interference of gravitropism with hydrotropism seems to be highly conserved.

### Diversity of the interaction between root hydrotropism and root gravitropism among natural Arabidopsis variants

In this study, two types of decreases in root hydrotropism under stationary conditions were found among natural Arabidopsis variants. One type was found in Cvi-0 and MNF-Pot-75, and root gravitropism and root hydrotropism were weaker than those in Col-0 (Fig. 2b, d). The other type was found in Kas-2 and C24, and root hydrotropism under stationary conditions was much weaker than that of Col-0, but root gravitropism did not significantly differ from that of Col-0 (Fig. 2b, d). The former type might have been caused by differences in common mechanisms between root hydrotropism and root gravitropism. As a mutant that reduces root gravitropism as well as root hydrotropism, a weak allele of *gnom* (*gnom*^*B/E*^) has been reported, although another allele of *gnom* (*gnom*^*miz2*^) was defective in root hydrotropism but not defective in root gravitropism (Geldner et al. 2004; Miyazawa et al. 2009). Brefeldin A (BFA), which inhibits the activities of guanine-nucleotide exchange factors, including GNOM, for ADP-ribosylation factor-type small GTPase and inhibits vesicle transport, also inhibits root hydrotropism as well as root gravitropism (Miyazawa et al. 2009). Therefore, there is a possibility that Arabidopsis natural variants that reduce root hydrotropism as well as root gravitropism have differences in vesicle transport systems from other natural variants.

The latter type, which reduces root hydrotropism but exhibits normal root gravitropism, is possibly caused by a specific reduction in root hydrotropism. It has been found that *miz1* and *gnom*^*miz2*^ mutants are defective in root hydrotropism but not in root gravitropism (Kobayashi et al. 2007; Miyazawa et al. 2009). Additionally, it is possible that mutations that reduce ABA or ABA responses decrease root hydrotropism but do not reduce root gravitropism (Antoni et al. 2013; Dietrich et al. 2017; Miao et al. 2021; Takahashi et al. 2002). Therefore, it is possible that polymorphisms in the ABA-related genes affect the expression of root hydrotropism in the latter type of Arabidopsis natural variants.

### Biological significance of the change in the interaction between root hydrotropism and root gravitropism

In this study, we screened 217 accessions of Arabidopsis by root hydrotropism under stationary conditions and found that the strength of hydrotropism is very diverse, but gravitropism is relatively conserved. In the natural environment, the water potential gradient is diverse, and the direction of gravity is consistent. When the two directions are inconsistent, root hydrotropism and root gravitropism must interact to determine the growth direction of roots. The interference of root gravitropism with hydrotropism is relatively conserved, which may be related to the prioritization of taproot function. As opposed to growing toward water, primary roots must first grow deep in the soil to anchor the plant into the ground.

To express root gravitropism, the *IGT* gene family plays a role in early responses to gravity (Dardick et al. 2013; Taniguchi et al. 2017). A rice natural variant, IR64, which reduces root gravitropism by a 1-bp deletion in the *DEEPER ROOTING 1* (*DRO1*) gene belonging to the *IGT* gene family, does not develop a deep root system architecture (Uga et al. 2013). As a result, IR64 is thought to reduce drought tolerance and rice yield (Arai-Sanoh et al. 2014; Uga et al. 2013). Additionally, it has been shown that the *qSOR1* (*quantitative trait locus for SOIL SURFACE ROOTING 1*) gene, which is a *DRO1* homolog, loss-of-function mutant avoids saline soil in paddy fields by showing reduced root gravitropism and has improved rice yields (Kitomi et al. 2020). Similarly, the *miz1* mutant, which is defective in root hydrotropism and normal root gravitropism, does not develop an extensive root system architecture in wetter ground (Iwata et al. 2013). On the other hand, the *MIZ1* overexpression line shows enhanced root hydrotropism and develops normal root system architecture in wetter ground (Iwata et al. 2013). Therefore, it is likely that changes in the interaction between root gravitropism and root hydrotropism affect the development of root system architecture in soil to allow plants to adapt to water stress conditions. The further identification of genetic differences that cause diversity in root hydrotropism would be worthwhile to understand how plants genetically adapt to water stress conditions.

To identify polymorphisms responsible for differences among Arabidopsis natural variants, we conducted GWAS using the curvatures of root hydrotropism under stationary conditions of 217 accessions of Arabidopsis. The T-DNA mutant of *MYB52* gene that contains a significant nonsynonymous polymorphism in our GWAS expressed normal root hydrotropism under stationary conditions (Fig. 5). However, under clinorotation, the hydrotropic curvatures of *mby52* mutants were significantly enhanced (Fig. 5). It is possible that several polymorphisms affect root hydrotropism under stationary conditions; therefore knockout of one gene is not enough to produce a given phenotype under stationary conditions. In contrast, clinostat conditions would be more sensitive to detect enhancement of root hydrotropism than stationary conditions. Therefore, clinostat conditions seem to allow us to detect enhancement of hydrotropic responses in roots of *myb52* mutant. In addition, the hydrotropic curvatures of accessions that contain the nonsynonymous polymorphism of *MYB52* were greater than those of accessions that contain normal allele (Fig. S2). Therefore, the nonsynonymous polymorphism of *MYB52* seems to contribute to facilitating hydrotropic responses.

*MYB52* gene encodes a transcriptional factor that activates expressions of *PECTIN METHYLESTERASE INHIBITOR6* (*PMEI6*), *PMEI14*, and *SUBTILISIN-LIKE SER PROTEASE1.7* (*SBT1.7*) genes of which products are involved in seed coat mucilage demethylesterification (Shi et al. 2018). Therefore, it is likely that changes of cell-wall composition of *myb52* mutants as well as some natural variants containing the nonsynonymous polymorphism affect hydrotropic responses on clinostat. Because a significant nonsynonymous polymorphism was found in C-terminal side that is outside of the conserved MYB DNA binding domain in N-terminal side, the nonsynonymous polymorphism would affect other than DNA binding activities of *MYB52* gene products.

## Conclusions

In this study, we examined root hydrotropism among various Arabidopsis accessions and found diversity in root hydrotropism under stationary conditions among natural Arabidopsis variants. Many Arabidopsis accessions can express root hydrotropism under stationary conditions, although some Arabidopsis accessions, such as Cvi-0 and MNF-Pot-75 accessions, could not. However, removal of gravity responses by clinorotation facilitated root hydrotropism in the examined Arabidopsis accessions including MNF-Pot-75 accession. These results suggested that interference of gravitropism with hydrotropism is highly conserved, although the intensity of root gravitropism interference with root hydrotropism differs.

## Supplementary Information

Below is the link to the electronic supplementary material.

Supplementary file1 (PDF 1056 KB)
