# Supplementary material for: Diversity of root hydrotropism among natural variants of Arabidopsis thaliana
Source: J Plant Res. 2022 Sep 23;135(6):799–808. doi: 10.1007/s10265-022-01412-w (PMC10039817; doi:10.1007/s10265-022-01412-w)
Supplement: Supplementary file 1 — Supplementary file1 (PDF 1056 KB) [file 10265_2022_1412_MOESM1_ESM.pdf]

Electronic supplementary materials

Title: Diversity of Root Hydrotropism among Natural Variants of Arabidopsis

Authors: Boyuan Mao, Hiroki Takahashi, Hideyuki Takahashi and Nobuharu Fujii*

Graduate School of Life Sciences, Tohoku University

2-1-1 Katahira, Aoba-ku, 980-8577,

Sendai, Japan

Journal:
Journal of Plant Research

Corresponding author:

Nobuharu Fujii

Tel: +81-22-217-5717

E-mail: nobuharu.fujii.b7@tohoku.ac.jp

Content:

Table S1, Figs. S1-S4

Table S1 Accessions with their mean hydropic curvature

Accession ID	Name	**Hydropic curvature (degrees)**	Country
6085	Sparta-1	39.33	SWE
7288	Oy-0	50.58	NOR
8227	THÖ 03	28.69	SWE
6932	Ler-1	32.03	GER
6013	Eden-9	59.47	SWE
1062	Brösarp-15-138	45.09	SWE
8376	Sanna-2	57.25	SWE
5832	App1-16	40.79	SWE
6016	Eds-1	67.17	SWE
8256	Bå1-2	43.42	SWE
9363	EdJ 2	64.63	SWE
6071	Omn-5	43.29	SWE
6214	TFÄ 04	14.35	SWE
6220	TGR 01	28.55	SWE
9332	Bar 1	25.51	SWE
6064	Nyl-2	65.98	SWE
5784	Ty-1	65.77	UK
6203	TDr-18	32.79	SWE
6900	Bil-5	36.98	SWE
6163	TAA 14	28.21	SWE
9868	Moe-0	38.23	ESP
9321	Ådal 1	49.60	SWE
7353	Tha-1	76.25	NED
8230	Algutsrum	44.86	SWE
6189	TDr-2	40.80	SWE
6244	TRÄ 01	57.46	SWE
9388	Grön 14	66.48	SWE
1002	Ale-Stenar-64-24	42.30	SWE
8427	Ull2-13	51.13	SWE
15592	OOE3-1	31.80	AUT

Accession ID	Name	**Hydropic curvature (degrees)**	Country
8311	In-0	34.10	AUT
6184	TBÖ 01	48.97	SWE
6237	TOM 03	47.33	SWE
9557	IP-Moa-0	30.64	ESP
9336	Bön 1	41.56	SWE
9564	IP-Nog-17	34.70	ESP
6192	TDr-5	13.92	SWE
6074	Ör-1	60.57	SWE
6038	Hov3-5	26.10	SWE
8258	Bå4-1	27.31	SWE
5856	Dör-10	41.70	SWE
997	Ale-Stenar-56-14	26.30	SWE
9824	Bes-5	43.59	ESP
6210	TEDEN 03	36.23	SWE
6911	Cvi-0	-32.40	CPV
6172	TÅD 04	35.55	SWE
6096	T1060	41.59	SWE
9567	IP-Pal-0	70.05	ESP
9528	IP-Cal-0	48.55	ESP
6177	TÄL 03	51.61	SWE
6917	Fäb-2	48.66	SWE
9568	IP-Pan-0	58.44	ESP
9104	Lag1-6	36.67	GEO
9433	Nyl 13	26.78	SWE
6034	Hov1-7	2.89	SWE
6235	TOM 01	53.99	SWE
1552	Sku-30	32.79	SWE
6030	Grön-5	25.52	SWE
9427	Näs 2	47.17	SWE
6070	Omn-1	44.62	SWE
6009	Eden-1	37.81	SWE

Accession ID	Name	**Hydropic curvature (degrees)**	Country
6046	Löv-5	14.05	SWE
9899	Tau-0	58.78	ESP
6115	T580	20.50	SWE
6036	Hov3-2	25.76	SWE
6077	Rev-3	55.79	SWE
6169	TÅD 01	26.55	SWE
6969	Tamm-27	51.60	FIN
8259	Bå5-1	54.88	SWE
6043	Löv-1	48.05	SWE
9545	IP-Her-12	32.32	ESP
6944	NFA-8	16.82	UK
9991	Vash-1	98.23	GEO
9455	Ste 4	32.94	SWE
6284	TV-38	-1.56	SWE
6976	Uod-7	27.04	AUT
8354	Per-1	51.18	RUS
7025	Bl-1	14.65	ITA
8343	Na-1	16.90	FRA
6931	Kz-9	-19.84	KAZ
9620	Basta-2	43.96	RUS
9840	Dar-0	44.04	ESP
9878	Pee-0	21.99	ESP
8249	Vimmerby	37.35	SWE
9642	Rakit-3	27.09	RUS
2057	Map-42	42.13	USA
9867	Mie-1	-10.26	ESP
6076	Rev-2	31.33	SWE
8326	Lis-1	9.89	SWE
6413	Ull3-4	-4.83	SWE
9641	Rakit-2	47.15	RUS
768	Zal-1	25.39	KGZ

Accession ID	Name	**Hydropic curvature (degrees)**	Country
9632	Lebja-2	36.44	RUS
7382	Utrecht	27.53	NED
5718	UKID11	32.15	UK
9594	IP-Vdm-0	46.48	ESP
9535	IP-Coc-1	39.02	ESP
6967	Sq-8	22.29	UK
6987	Ak-1	9.01	GER
9597	IP-Vig-1	28.83	ESP
9595	IP-Vdt-0	47.45	ESP
5772	Set-1	28.79	UK
7307	Pn-0	46.55	FRA
6981	Ws-2	36.18	RUS
8424	Kas-2	6.45	IND
5349	UKSE06-639	21.34	UK
9421	Lan 1	49.91	SWE
9891	Sal-0	37.44	ESP
9886	Pru-0	59.27	ESP
6191	TDr-4	12.33	SWE
8337	Mir-0	13.08	ITA
7081	Co	35.27	POR
9636	Noveg-1	7.34	RUS
6979	Wei-0	28.93	SUI
9836	Cod-0	38.00	ESP
8387	St-0	36.47	SWE
9621	Basta-3	49.08	RUS
6971	Ts-5	59.44	ESP
7378	Uk-1	14.81	GER
7298	Pi-0	-0.07	AUT
6945	Nok-3	49.88	NED
7396	Ws-0	53.82	RUS
9091	Nar-5	16.84	AZE

Accession ID	Name	**Hydropic curvature (degrees)**	Country
9873	Ndc-0	9.46	ESP
6188	TDr-1	44.62	SWE
5651	UKNW06-488	42.88	UK
6908	CIBC-5	-0.70	UK
4857	UKSW06-257	49.41	UK
6243	Tottarp-2	32.88	SWE
9705	Choto-1	61.65	BUL
6984	Zdr-1	-8.36	CZE
7067	Ct-1	6.93	ITA
8297	Ge-0	20.71	SUI
6915	Ei-2	31.04	GER
9897	Smt-1	8.08	ESP
9075	Lerik1-4	9.08	AZE
9834	Cho-0	6.87	ESP
9946	Mer-6	17.06	ESP
9841	Ees-0	8.47	ESP
7255	Mh-0	5.61	GER
6073	ÖMö1-7	40.69	SWE
5837	Bor-1	65.39	CZE
7418	Zu-1	7.04	SUI
7520	Lp2-2	49.02	CZE
6970	Ts-1	34.45	ESP
9888	Pva-1	36.72	ESP
9638	Noveg-3	21.18	RUS
801	KYC-33	18.60	USA
7383	Van-0	36.57	CAN
9904	Vas-0	-17.69	ESP
6042	Lom1-1	47.21	SWE
6951	Pu2-23	33.66	CZE
7282	Or-0	-8.92	GER
7373	Tsu-0	50.82	JPN

Accession ID	Name	**Hydropic curvature (degrees)**	Country
9831	Cas-0	36.38	ESP
350	TOU-A1-88	18.25	FRA
7127	Est	8.48	GER
8366	Rd-0	26.76	GER
9559	IP-Mon-5	65.79	ESP
991	Ale-Stenar-41-1	39.18	SWE
9586	IP-Tam-0	30.25	ESP
5776	UKID71	32.53	UK
9078	Lerik1-7	7.15	AZE
7160	Gre-0	62.99	USA
8312	Is-0	49.74	GER
5741	For-2	21.08	UK
5717	Bra-1	40.74	UK
7111	Edi-0	27.12	UK
9912	CIRY-13	44.36	FRA
7404	Wc-1	19.76	GER
9902	Usa-0	12.09	ESP
6101	T1120	29.94	SWE
9058	Västervik	0.86	SWE
1872	MNF-Pot-75	-19.25	USA
9637	Noveg-2	15.74	RUS
7003	Bs-1	-1.72	SUI
6008	Duk	3.02	CZE
5768	UKID63	41.11	UK
9817	Ace-0	-1.65	ESP
9789	Obh-13	3.20	GER
7287	Ove-0	24.86	GER
9089	Nar-3	14.64	AZE
9758	Altai-5	6.12	CHN
7477	WAR	19.48	USA
7117	El-0	65.56	GER

Accession ID	Name	**Hydropic curvature (degrees)**	Country
6940	Mz-0	26.17	GER
9825	Boa-0	0.74	ESP
2317	Ste-40	36.99	USA
9530	IP-Car-1	-6.23	ESP
7036	Bu-0	-0.25	GER
5395	UKNW06-102	44.30	UK
6s929	Kondara	67.18	TJK
9860	Lum-0	29.30	ESP
6927	Kno-10	19.17	USA
7217	Lm-2	40.20	FRA
9835	Cir-0	-12.66	ESP
7372	Tscha-1	25.31	AUT
9565	IP-Orb-10	-7.33	ESP
7186	Kn-0	46.52	LTU
2171	Paw-26	5.84	USA
6201	TDr-16	3.29	SWE
6898	An-1	17.83	BEL
7126	Es-0	46.50	FIN
6975	Uod-1	18.86	AUT
7417	Zu-0	35.75	SUI
9985	ICE29	30.62	BUL
6909	Col-0	39.77	USA
6982	Wt-5	32.38	GER
7058	Bur-0	39.36	IRL
8214	Gy-0	27.52	FRA
7273	No-0	22.53	GER
8420	Kelsterbach-4	14.16	GER
9070	Xan-6	1.87	AZE
1006	Ale-Stenar-77-31	24.26	SWE
6041	Lis-3	9.36	SWE

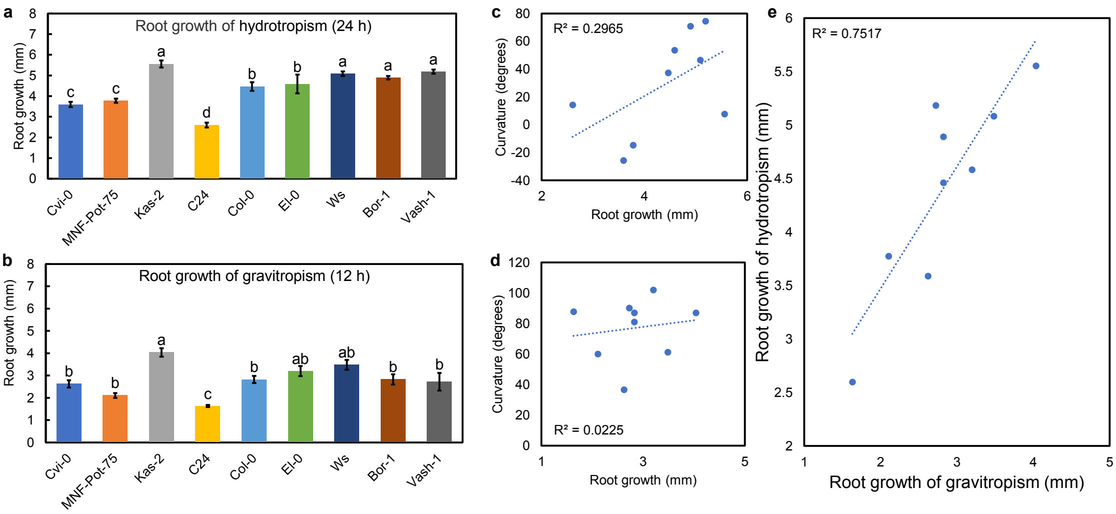

Fig. S1 Relationship between root growth and root curvature during root hydrotropism and root gravitropism of representative natural variants of Arabidopsis.

a Root growth during hydrotropism for 24 h. **b** Root growth during gravitropism for 12 h. Each data point is the average of three independent experiments that were conducted using 8-10 individuals per experiment. Error bars represent SEs. Different letters indicate statistically significant differences ($P < 0.05$) by Tukey's honestly significant difference (HSD) test. **c** Scatter plot of root growth (x-axis) and root curvature (y-axis) during hydrotropism for 24 h. **d** Scatter plot of root growth (x-axis) and root curvature (y-axis) during gravitropism for 12 h. **e** Scatter plot of root growth during gravitropism for 12 h (x-axis) and root growth during hydrotropism for 24 h (y-axis). The R^2 value and the regression equation are shown.

Gene	Codon	Position	Strip chart	Amino
AT1G17950 *MYB52*	cCt/cGt	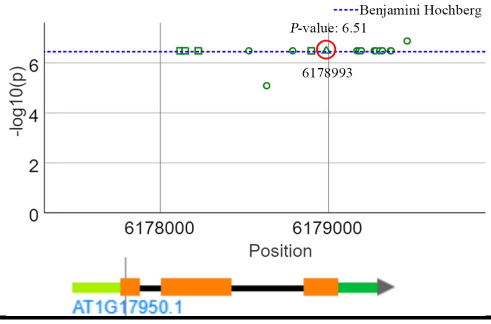	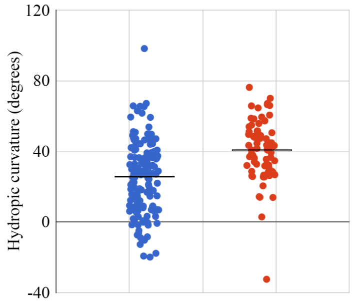	P227R

Fig. S2 Significant nonsynonymous polymorphisms. In the "Position" column, nonsynonymous SNPs (△) and synonymous SNPs (□) are shown in the upper part. The red circle indicates a nonsynonymous SNP that is described in the row. The *y*-axis represents -log10(*P*) values. The *x*-axis is the positions on the chromosome. In the lower part, the gene model is shown. In the "Strip chart" column, hydrotropic curvatures under stationary conditions of the WT (Col-0 type) alleles and altered alleles are shown by blue and red dots, respectively. Black horizontal lines indicate the averages.

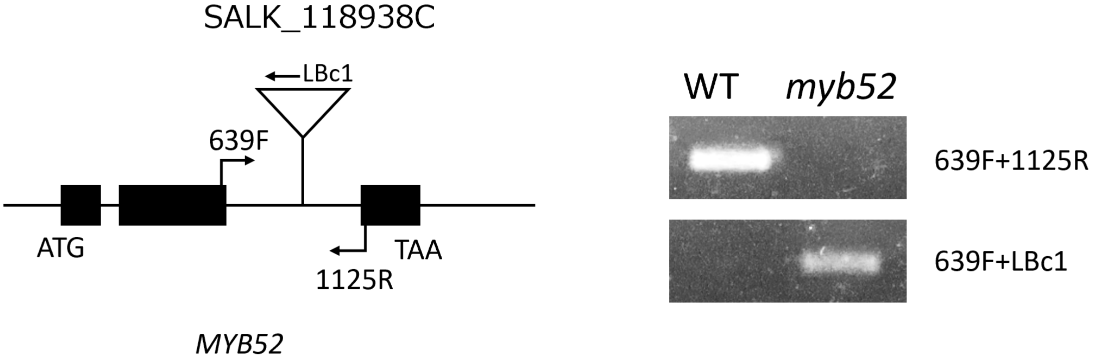

Fig.S3 Location of T-DNA insertion in the *MYB52* gene in Arabidopsis thaliana. Left, Schematic representation of *MYB52* gene. Boxes represent exons. A triangle and arrows indicate the position of T-DNA insertion and primers, respectively. Right, Genotyping of the T-DNA insertion into *MYB52* gene in *myb52* mutant (SALK_118938C) by PCR and agarose gel electrophoresis. The primers used were as follows: 639F (5'- CCGGAAAGATCGGGTTCAGA-3'); 1125R (5′-TCCATCGGTCGTTTAGTTTGGT-3'); LBc1, T-DNA left border primer (5'-CTTGCTGCAACTCTCTCAGG-3').

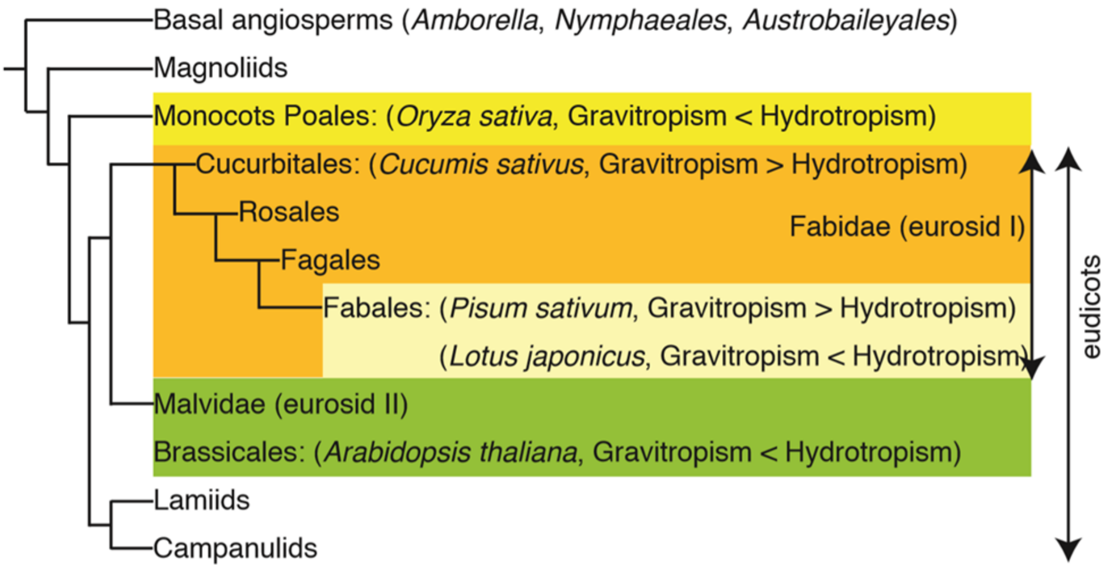

Fig. S4 Phylogenetic relationship of the interaction between root hydrotropism and root gravitropism. The comparison of gravitropism and hydrotropism in plant species already revealed is shown within the brackets. Simplified from the angiosperm classification of the Angiosperm Phylogeny Group (APG) IV (2016) is shown.

APG IV (2016) An update of the Angiosperm Phylogeny Group classification for the orders and families of flowering plants: APG IV. Bot J Linn Soc 181:1–20. doi.org/10.1111/boj.12385
